# Editorial: Cardiovascular neuromodulation: mechanisms and therapies

**DOI:** 10.3389/fcvm.2023.1214496

**Published:** 2023-05-23

**Authors:** Deborah Hunt, Marco Mongillo, Marianna Meo, Tania Zaglia, Khaled Qanud

**Affiliations:** ^1^ College of Nursing and Public Health, Adelphi University, Garden City, NY, United States; ^2^ Biomedical Sciences, University of Padova, Padova, Italy; ^3^ Boston Scientific, Boston Scientific, Kerkrade, Netherlands; ^4^ Bioelectronic Medicine, Feinstein Institutes for Medical Research, Manhasset, NY, United States

**Keywords:** heart, neuromodulation, autonomic nervous system, heart failiure, hypertension, vagus nerve stimulation

**Editorial on the Research Topic** Cardiovascular neuromodulation: mechanisms and therapies

High cardiovascular disease (CVDs) prevalence is projected to impact a large population across the world (1). Future therapeutic development efforts should take these estimates into account and provide new treatment modalities.

Modern neuromodulation therapies are an emerging non-pharmacological approach for the treatment of several disease conditions in basic research and clinical studies. The principial basis is to reduce or enhance, selectively, the altered neural activity that determined by the pathophysiological mechanisms, using neuro-medical devices (2). The main attractive subject is the autonomic nervous system (ANS) which maintains the body homeostasis, and the disruption of its integrity cornubites to the development and progression of many diseases including those affecting cardiovascular system and the immune components (3, 4).

**Figure 1:**
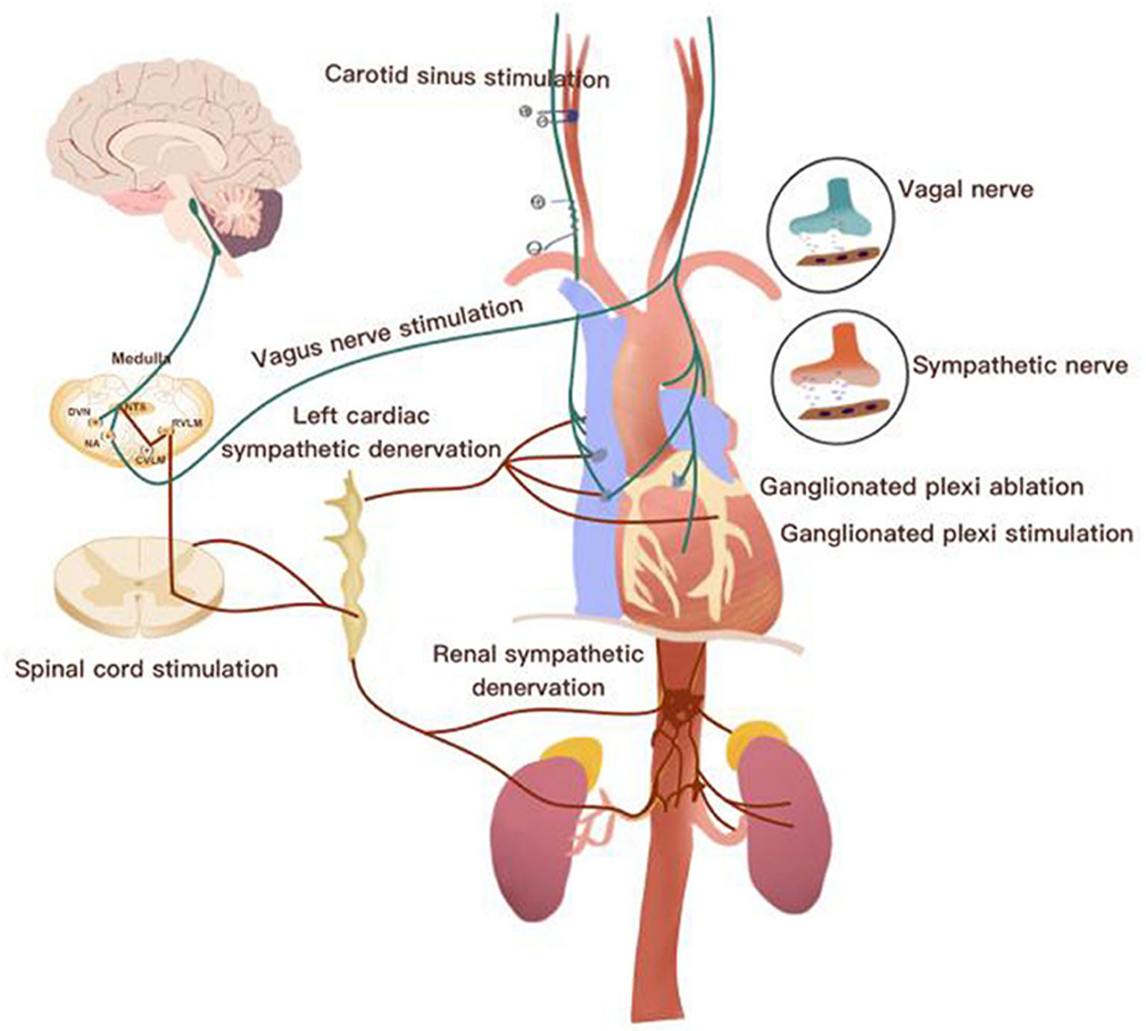
Overview of the neurocardiac axis and invasive neuromodulation approaches [Adapted from Chen et al., (10)].

The cardiac autonomic nervous system provides a closed-loop control of the heart and vascular system through a rich highly organized neural network composed of the brainstem, extracardiac sympathetic ganglia, vagus nerve and the intrinsic cardiac nerves system. Dysregulation at any level could lead ANS imbalance and, also, could trigger the chronic inflammatory process (5–7). Initially, dysregulation of the ANS may serve as a compensatory mechanism to maintain blood pressure and cardiac output in response to a cardiovascular insult, such as myocardial infarction and heart failure that associated with exaggerated sympathetic overflow and reduction of parasympathetic tone. However, if this dysregulation is not relieved by therapy, it becomes maladaptation and can lead to the development of a wide range of cardiovascular and non-cardiovascular conditions over time (8, 9). The beneficial effects of cardiac-brain-axis modulation were highlighted in multiple pre-clinical and clinical studies. For example, these studies included: renal denervation (RDN) to treat resistant hypertension, restore the baroreflex tone to treat orthostatic hypotension (OT), cardiac sympathectomy to suppress arrhythmias and vagus nerve stimulation (VNS) to reduce the progression of HF (Figure 1) (10–15).

In this issue the review by Ottaviani et al. has explored in detail the histological structure and the physiological role of vagus nerve, as the main tool to provide cardiovascular neuromodulation, and presented preclinical studies aimed at overcoming VNS limitations through optimization of anatomical targets, development of novel neural interface technologies, and design of efficient VNS closed-loop protocols. The interesting findings that were documented in the manuscript by Rodrigues et al. who measured blindly the effects of acute and short-term transcranial direct current stimulation (tDCS) sessions on blood pressure and autonomic modulation in RHT subjects and showed a reduction in the central blood pressure. The heart rate variability (HRV) as a measure of ANS balance was reduced in association with post MI arrhythmic events which increased the mortality in the study that was conducted by Pizzo et al. There was also a U-shaped association between HRVI and mortality in hemodialysis AF patients as found in the data from Braunisch et al. The sophisticated study by Neely et al. examined whether macrophages could drive the sympathetic phenotype in Spontaneously Hypertensive Rats (SHR), before animals develop high pressure; their findings showed that macrophages can be potent enhancers of sympathetic neuronal calcium responsiveness and plays a role in peripheral sympathetic hyperactivity observed in the initial stages of hypertension.

Cardiovascular neuromodulation is an emerging field with ongoing research and clinical trials to investigate its safety and efficacy in various cardiovascular conditions. It has the potential to offer new treatment options for patients with cardiovascular conditions that fail to respond to the traditional therapies. However, further research is needed to fully understand its mechanisms of action and long-term outcomes.
